# Challenges underlying radiology’s research problem

**DOI:** 10.1186/s13244-026-02219-2

**Published:** 2026-04-10

**Authors:** Luis Martí-Bonmatí, Daniel Pinto Dos Santos, Diana Veiga-Canuto, Christian Loewe

**Affiliations:** 1 Medical Imaging Department. Hospital Universitario y Politécnico La Fe, Valencia, Spain; 2 Biomedical Imaging Research Group. Health Research Institute, Valencia, Spain; 3 Department of Radiology, University Medical Center Mainz, Mainz, Germany; 4 Department of Bioimaging and Image-Guided Therapy. Medical University of Vienna, Vienna, Austria

**Keywords:** Research, Radiology, Training

## Abstract

**Abstract:**

Radiology faces structural, cultural, and systemic challenges that threaten the sustainability and quality of its research. This article critically examines the complex factors shaping radiological research engagement, emphasizing the need to redefine academic value systems within the specialty.

Radiology training remains largely generalist, limiting disease-specific clinical insight, while rapid technological advances have shifted research toward engineering and computational fields, eroding radiology’s clinical ownership of imaging innovation. Multidisciplinary collaboration among radiologists, clinicians, physicists, and data scientists is essential to maintain clinically meaningful and translational research. Establishing disease-focused groups within professional societies will surely promote subspecialized expertise and guidance. Training and mentorship gaps further impede progress. Traditional curricula provide limited research exposure, while early subspecialization, structured mentorship, and integrated research training during residency are proposed to foster clinical excellence and scientific engagement. Increasing productivity demands, together with unprecedented digital workflow pressures, contribute to cognitive overload and burnout. These factors discourage participation in research. Institutional reforms ensuring protected research time, equitable recognition of academic work, and improved cognitive ergonomics are vital for innovation. Funding constraints and limited grant-writing skills reduce competitiveness, as imaging research often struggles against therapeutic fields. Developing collaborative research networks and scientific writing training can enhance funding success. Finally, quantity-driven academic metrics promote low-value publications. A shift toward value-based evaluation—prioritizing clinical relevance, methodological rigor, and collaboration—is therefore needed.

Radiology must realign its academic structures to sustain high-quality research and reaffirm its dual role as a clinical cornerstone and scientific innovator. The Cancer Image Europe (EUCAIM) infrastructure facilitates collaboration among radiologists, clinicians, researchers, and innovators, representing a valuable opportunity for radiologists in training to engage in research.

**Critical relevance statement:**

Radiology faces structural, cultural, and systemic challenges that threaten the sustainability and quality of its research. This article critically examines the complex factors shaping radiological research engagement, emphasizing the need to redefine academic value systems within the specialty.

**Key Points:**

- Radiology must overcome structural and cultural barriers to sustain impactful research.
- Multidisciplinary collaboration and disease-focused training are key to clinical innovation.
- Reforming evaluation systems and protecting research time are essential for progress.

**Graphical Abstract:**

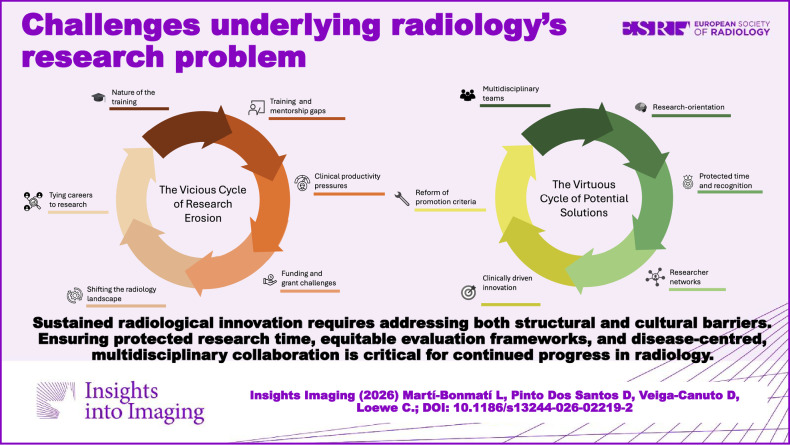

## Introduction

Imagine the following scenario: You are the head of the radiology department, looking to hire a new colleague or decide on a promotion. Your schedule is—as always—tight, but a decision needs to be made. You have several applications on your desk, all of similar age and background; not much stands out, except that some candidates list varying numbers of peer-reviewed publications in their CV. Would you dive deep into their publications and carefully weigh the quality of each manuscript? Or would the absolute number of publications be a good enough indicator for scientific potential? Will you definitively prefer a candidate with a larger knowledge of research and critical thinking? Is research really relevant for the head of departments as a competitive advantage? Obviously, the answers cannot be universal. They vary according to the institutional context (community versus large regional/tertiary hospitals), the ownership model (private versus academic), and local workforce constraints, including radiologist availability.

Generally speaking, there is a broad consensus among radiology leaders that research training is fundamental for maintaining sustained innovative clinical activity at least within academic and referral medical departments [1]. During training, trainees should learn how to assess research quality, recognize biased and poorly powered research [2]. But also, experience hands-on participation in research provides them with a foundation to understand research methodology, evaluate clinical impact, and improve their imaging data interpretation. Ideally, this should be done progressively as part of a structured curriculum with mentorship and collaborative interdisciplinary work [1, 3]. This research culture is vital for radiology, both to maintain the quality of practice and to contribute to clinical innovation and guideline making [4].

Although some radiologists engage in research, over the last years several heads of academic departments have voiced concerns that either participation in research is lacking among their colleagues, or the quality of research output has decreased. We must therefore ask ourselves whether this observation is merely anecdotal or if underlying structural and cultural factors might be undermining the otherwise commendable efforts to pursue research with high-quality standards [1]. At the same time, it is important to acknowledge that several of these challenges, such as burnout, increasing cognitive workload, and metric-driven academic evaluation, are not unique to radiology but reflect broader trends across academic medicine. Recognizing this wider context helps clarify which issues are systemic and which may be disproportionately amplified within the radiology community [5].

**Fig. 1:**
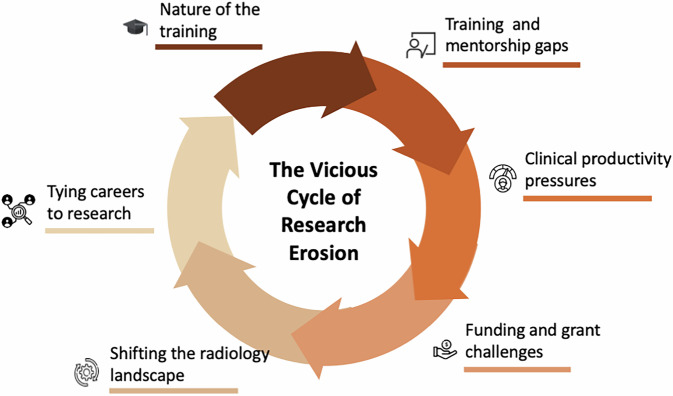
The vicious cycle of research erosion in radiology. Interrelated factors contributing to the progressive decline of research activity are depicted, including the nature of training, gaps in research training and mentorship, increasing clinical productivity pressures, funding and grant-related challenges, shifts in the radiology landscape, and the growing tendency to link career progression primarily to research output. Together, these elements form a self-reinforcing cycle that undermines sustained research engagement

In the authors’ view, although robust statistical evidence may still be lacking, several plausible factors could be more pronounced in the radiology community than in other specialties and therefore warrant further investigation. The following sections outline several key issues and potential strategies that may directly or indirectly influence the relevance, visibility, and standing of radiology within today’s highly competitive clinical and research environments (Fig. 1). Although these challenges are presented thematically, they are deeply interconnected. Generalist training, for instance, may limit early disease-specific expertise, which in turn weakens the competitiveness of grant applications. This reinforces dependency on high-volume clinical activity, further intensifying cognitive workload and contributing to burnout [6]. This cascading relationship underscores that radiology’s research difficulties do not arise from isolated factors but from a mutually reinforcing system.

## Nature of the training and fundamental shifts in radiology

Training in radiology is often general in nature, as the needed technical and clinical skills are huge. Therefore, training is limited in terms of in-depth understanding of specific pathologies and treatments. While a well-trained radiologist may be skilled at detecting a wide range of conditions, the rapid advancements in treatment and disease management mean that radiologists often lack deeper knowledge of the corresponding clinical pathways and treatment options [7]. Consequently, radiology is often perceived as a consultative imaging service supporting other medical specialties rather than a primary specialty driving clinical decisions [2]. Furthermore, the rapid progress in biomedical engineering, computer science and artificial intelligence has shifted many research questions in radiology toward technological or methodological domains [8], often led today by engineers or physicists. Although direct cross-specialty comparisons with engineering or computer science are lacking, published analyses show that NIH funding for radiology has grown over the past decade, even outpacing other clinical departments in R01 and R01-equivalent grants [9, 10]. Bibliometric studies of imaging-focused AI research reveal that a substantial portion of highly cited work is published in engineering and computer science journals, often led by investigators from these fields, reflecting a gradual shift of imaging research toward computational and engineering domains [11, 12]. This trend is further supported by rapid growth in informatics and imaging biomarker research within radiology [13], and a much broader, more multidisciplinary mix.

To maximize radiologist’s clinical relevance and minimize biases, broad multidisciplinary research teams are essential [14, 15]. One potential approach is the development of working groups organized by pathology or disease-focused topics within regional communities or professional societies, where senior radiologists participate and mentor junior radiologists interested in these areas. Such network structures, even been virtual with remote engagement, would facilitate knowledge transfer, foster focused expertise, and strengthen the clinical impact of radiology research [15]. Fostering collaboration and promoting innovation are essential for advancing the quality, competitiveness, and clinical significance of radiology research [3]. These structural training limitations have downstream effects on mentorship and early academic development.

## Training and mentorship gaps, and the need for subspecialization

Radiologists are not always trained in large academic or reference centers. This may further limit early exposure to clinically oriented or research-focused training in well-defined multidisciplinary teams with active mentorships [1]. Even more, training programs still adhere to the traditional idea that residents must be educated in the entire field of radiology, with residency curricula fully packed, leaving limited time for research and focused developments [16]. At the same time, there is an inherent tension between preserving the broad generalist diagnostic foundation required for safe and competent clinical practice and promoting earlier subspecialization to enable deeper, disease-focused research. Acknowledging this balance is essential when designing training pathways that aim to strengthen both clinical performance and academic development.

Importantly, the European Society of Radiology (ESR) recognizes research and evidence-based medicine as core components of radiology training. The ESR curriculum defines essential research knowledge, skills, and competences, including scientific methodology, study design, basic biostatistics, critical appraisal of the radiological literature, and the ability to plan and conduct research under supervision. In addition, ESR subspecialization curricula promote active participation in radiological research projects, reinforcing research as an integral component of advanced clinical training [17].

Building on this, more focused and in-depth training on specific clinical aspects, earlier subspecialization (whether along clinical or technological pathways), access to personalized mentorship opportunities [18, 19], and the establishment of multidisciplinary research groups may be needed, not only to provide better clinical services but also to foster more meaningful research [2, 8, 14, 20]. Radiology residency research tracks can be implemented as dedicated research pathways for those willing to become academic radiologists [16]. A proposed pathway is depicted in Table 1. Identifying and engaging with an effective mentor is a critical determinant of a researcher’s growth, facilitating knowledge transfer, skill development, and academic maturation [19]. In this context, radiology research fellowships—such as those offered by the European School of Radiology (ESOR)—provide valuable opportunities for young radiologists to strengthen their academic competitiveness through immersive subspecialty training, close mentorship under experienced physicians, and participation in intensive, research-oriented learning experiences [21, 22]. Even when trainees are motivated to pursue research, the increasing demands of clinical productivity further restrict the time and cognitive space needed for academic engagement.

**Table 1:** Proposed integrated research pathway during radiology residency

Step	Protected research time	Main goal	Key outputs
1	Low (0.1–0.2 FTE)	Research literacy and orientation	Structured journal club critiques; shortlist of topics; mentor match; mini-proposal
2	Low (0.1–0.2 FTE)	Applied methods and project build	IRB submission; curated dataset; pilot analysis; poster/local presentation
3	High (0.5–1.0 FTE)	Dedicated research block	Data collection completion; primary analysis; abstract to major meeting; first manuscript draft
4	Low–moderate (0.1–0.2 FTE)	Translation and grant/career prep	Manuscript submission(s); grant proposal (institutional/seed/national); leadership/teaching role
5 (opt)	Variable	Advanced/subspecialty research	Multi-center work; major grant resubmission; high-impact publication; faculty transition plan

Protected research time increases during dedicated research blocks while maintaining clinical responsibilities. Each stage develops core competencies, from research literacy and project design to data collection, analysis, manuscript preparation, and grant writing. Key outputs indicate typical milestones, including institutional review board submission, dataset curation, abstracts, manuscripts, and grant proposals, providing a structured roadmap to support residents pursuing academic and clinically meaningful research careers

*FTE* full-time equivalent, *IRB* institutional review board, *S* step

## Clinical productivity pressures and generational expectations

Unlike many other medical specialties, where maximum workload is defined by processes that include consultation with patients and mainly manual tasks, the shift from film-based radiology to fully digitized workflows has led to a massive boost in productivity for radiologists, if productivity is defined solely on reports per time unit, as it is in most volume-based healthcare settings today. This has led to an ever-increasing mental workload with a consequent risk of burnout among radiologists. Add to this the fair expectation that some form of work-life balance must be found, and it is obvious that for some radiologists, there is a strong individual incentive to not engage in research activities at all [1] so as not to add further cognitive burden and potential for mental exhaustion [16].

The solution to this is not an easy one, but it must include protected time [23] based on capabilities and achievements and recognizing previous research activities—such as participation in grants and publications—as equally important professional activities [8, 22]. While implementing protected research time may appear costly or challenging, it should be framed as a critical investment that can enhance innovation, maintain the specialty’s clinical relevance, and prevent the commoditization of radiology. Furthermore, cognitive load in radiology needs much more dedicated attention, as besides being detrimental to potential research activities, it might be a major contributor to diagnostic errors in the performance of radiology. This erosion of protected time and cognitive bandwidth affects grant competitiveness and long-term academic growth.

## Funding and grant challenges

Funding agencies frequently prioritize research that is directly connected to disease mechanisms or therapeutic interventions, while imaging-focused studies often find themselves competing with projects rooted in engineering and computer science. Consequently, radiology research faces structural disadvantages in securing competitive funding. Moreover, grant writing remains a significant challenge for many radiology departments, reflecting limited institutional support, interdisciplinary complexity, and a historical underemphasis on research-oriented training within the field [24, 25]. As a result, grant reviewers may not always fully appreciate the translational potential of medical imaging studies and derived data.

A potential solution would be to establish networks of experienced researchers with complementary expertise and shared objectives to strengthen proposals and highlight clinical relevance.

## Shifting the radiology research landscape

Radiology is undergoing a rapid and profound transformation driven by advances in imaging biomarkers, radiomics, and artificial intelligence. These technologies have led to a marked increase in imaging-focused research and publications, elevating the field’s prominence within the paradigm of data-driven precision medicine. As analytical models and quantitative imaging methods continue to evolve in complexity, it is imperative to ensure that these innovations remain clinically meaningful and anchored in patient care, diagnostic accuracy, and therapeutic decision-making.

Sustaining the link between technological innovation and translational clinical impact requires radiologists to remain proficient in both computational methodologies and their clinical applications. This expertise is essential for radiology to reach its full potential in precision healthcare [26].

## Tying career opportunities to research

Quality of research is notoriously difficult to measure and ultimately might not even be rewarded appropriately. It might have been easy to determine the impact of Wilhelm Conrad Roentgen’s discoveries and his individual contribution at the time, but if, for example, we think about the thousands of people collectively working on the CERN’s (*Conseil Européen pour la Recherche Nucléaire*) Large Hadron Collider, it might be much more difficult. And while we cannot comment on how career promotions are determined at the CERN, it is known that in many countries career advancements in academic medicine are often tied to the number of published papers as opposed to the quality of published papers—just like the supposed quality of a scientific journal or an individual publication is measured in the number of citations as opposed to its clinical impact or methodological rigor. This has led to the fact that there is a strong systemic incentive—especially for young researchers—to engage in quick-to-publish but ultimately clinically useless projects rather than engaging in deep-dive, long-term projects with a multidisciplinary team where the individual might not even be first or last author [27]. In theory, editors and reviewers should be able to prevent such useless research to be published, but experience shows that this is not the case and ultimately almost all papers written end up being published somewhere—especially since editors and reviewers in radiology for the most part suffer from all the effects mentioned above (cognitive overload, generalist vs. subspecialized knowledge and incentive to work through many papers shallowly vs. few papers deeply). In addition, meaningful reform of promotion criteria and evaluation metrics is inherently constrained by institutional governance structures. These systems typically extend beyond radiology, involving university-wide policies, hospital administration, and national frameworks, which may make any substantive change both slow and institutionally complex.

**Fig. 2:**
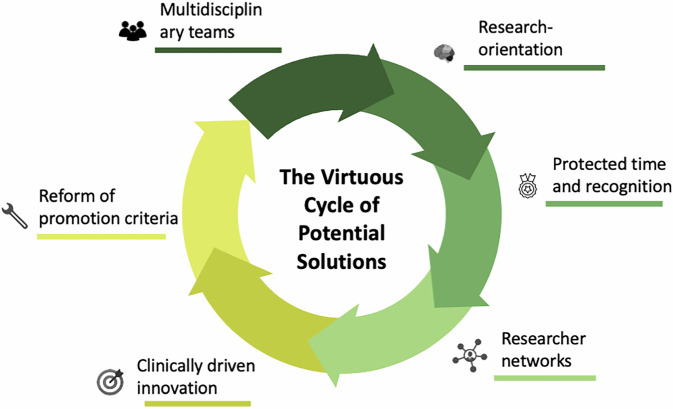
A virtuous cycle of potential solutions to strengthen radiology research. Key elements that may counteract research erosion are illustrated, including multidisciplinary collaboration, a research-oriented culture, protected time and appropriate recognition, researcher networks, innovation driven by clinical relevance, and reform of academic promotion criteria. The interaction of these factors promotes impactful and sustainable research careers

Addressing this challenge is complex and may even prove unattainable without profound systemic reform (Fig. 2). Nevertheless, radical and comprehensive changes are urgently required to transition from volume-based research—often lacking in clinical applicability—to value-based research that prioritizes scientific rigor, reproducibility, citations and genuine clinical relevance. For authors, combining the i10-index (the number of publications with at least 10 citations each), the h-index (the largest number h such that h papers have ≥ h citations), and the number of funded research projects with active participation provides a reasonable balance between quality and productivity for evaluating radiologist performance when looking to hire a new colleague or decide on a promotion.

## Conclusions

The decline in reading and performing research leads to a poorer understanding of reality and creates a new opportunity for professional neglect. The traditional negative ‘publish or perish’ dilemma should be reinterpreted in radiology as a positive call to continuously disseminate meaningful advances and improvements. Otherwise, the profession itself risks perishing.

Research remains a cornerstone of radiology’s progress, inspiring radiologists and shaping the profession through continuous innovation, translational application, and the advancement of imaging science. Beyond its technical achievements, radiology must now engage in a broader societal and academic dialogue about its future direction as both a medical and scientific discipline. The ongoing discussion on value in healthcare should equally encompass the value of research itself.

Just as we devote time and attention to interpreting an individual imaging study to ensure the best outcome for a single patient, we must also commit to long-term, rigorous, and complex research that can transform patient care in the future. Fostering genuine curiosity, creating protected time for research, and improving career opportunities for young radiologists and trainees are essential steps in this process. Yet, achieving these goals remains a formidable challenge in an increasingly fast-paced environment where most key performance indicators prioritize clinical throughput over scholarly contribution. The Cancer Image Europe (EUCAIM) infrastructure facilitates collaboration among radiologists, clinicians, researchers, and innovators, and represents a valuable opportunity for radiologists in training to engage in research, from individual PhD projects to large international consortia [28].
